# Temperature as a Climate Variable in Dengue Epidemiological Studies

**DOI:** 10.1590/0037-8682-0481-2025

**Published:** 2026-04-17

**Authors:** Tatiane Fernandes Portal de Lima Alves da Silva, Walter Massa Ramalho, Henry Maia Peixoto, Marco Aurélio de Valois Correia, André Luiz Sá de Oliveira

**Affiliations:** 1 Ministério da Saúde, Secretaria de Vigilância em Saúde e Ambiente, Departamento de Articulação Estratégica de Epidemiologia e Vigilância em Saúde, Brasília, DF, Brasil.; 2 Universidade de Brasília, Faculdade de Medicina, Núcleo de Medicina Tropical, Brasília, DF, Brasil.; 3 Universidade de Pernambuco, Programas de Pós-Graduação em Hebiatria e em Educação Física, Recife, PE, Brasil.; 4 Instituto Aggeu Magalhães, Núcleo de Estatística e Geoprocessamento, Recife, PE, Brasil.

**Dear Chief Editor of the *Journal of the Brazilian Society of Tropical Medicine*:**

Considering the influence of climatic variables on dengue incidence^1^ ^,^ ^2^ and in light of the findings of two recent articles published in this journal, namely, “The greatest Dengue epidemic in Brazil: Surveillance, Prevention, and Control”^3^ and “Climatic variables associated with dengue incidence in a city of the Western Brazilian Amazon region”^4^, this letter explores the role of temperature in the ongoing debate. It does not intend to exhaust the topic or replace broader analytical approaches.

 In the past two years, the number of days with temperatures above historical averages has increased. Coupled with hot days and summer rains, there has been an increase in vector-borne diseases^5^, including dengue, in Brazil and worldwide^2^ ^,^ ^5^ ^,^ ^6^. Surveillance, prevention, and control actions targeting *Aedes aegypti*, the main transmitting vector of the dengue virus (DENV) in Brazil, rely on epidemiological and predictive studies that incorporate climate data, among other variables^2^ ^,^ ^6^ ^-^ ^8^.

Gurgel-Gonçalves et al.^3^ discuss rising temperatures as a key driver of Brazil’s unprecedented dengue epidemic in 2024 and its expansion into areas previously considered less favorable for sustained transmission, including higher-altitude regions. Dengue has a complex and multifactorial dynamic that arises from interactions between environmental, socioeconomic, behavioral, immunological, and biological factors. The role of environmental factors, which are determinants of vector abundance, has intensified in recent years^5^. Well-coordinated and effective governmental and intergovernmental actions are required to mitigate the impact of these factors^9^.

Increased temperatures shorten the developmental cycle of *Aedes aegypti* (egg to adult) and the extrinsic incubation period of the virus^5^ ^,^ ^10^. Therefore, changes in environmental average temperatures may affect the disease cycle, ultimately leading to increased case numbers^5^. However, this effect may be reversed at extreme temperatures^10^.

Mosquito eggs and larvae rarely survive at temperatures above 40°C, a critical threshold for modeling purposes^11^. Accordingly, Duarte et al.^4^ focused on average temperature and its effect on dengue incidence. They found that a 1°C increase in the minimum average monthly temperature around 21°C, and in the adjusted average temperature (mean of minimum and maximum, approximately 25°C), was associated with a 54% increase in monthly dengue incidence. Conversely, each 1°C rise in maximum temperature (approximately 32°C) was associated with impaired transmission dynamics.

Our group estimated the dengue incidence rates in Brazilian municipalities in 2013 and 2024 using data from the Notifiable Diseases Information System (Sinan, in the Portuguese acronym). To explore spatial patterns, we calculated a bivariate Moran’s Global Index using Geoda and QGIS to examine the spatial dependence and correlation between municipal dengue incidence and average temperatures according to the Köppen climate classifications^12^.

**FIGURE 1:**
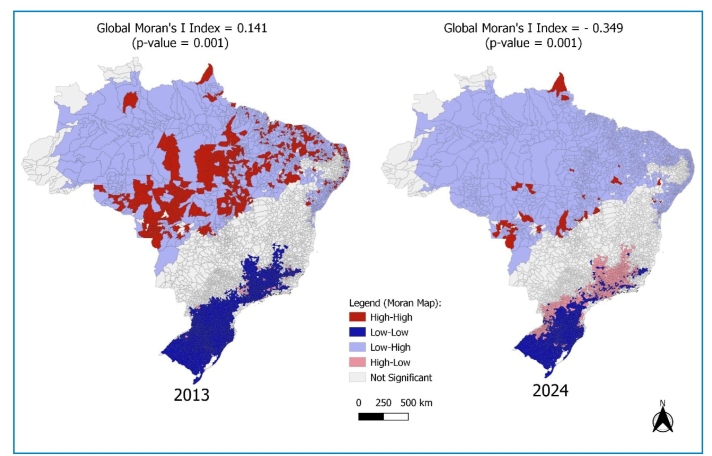
Bivariate Moran Map of dengue incidence rates in 2013 and 2024 and the climate variable of average temperature, according to Köppen^12^. The Global Moran’s Index was 0.141 in 2013 (P = 0.001) and −0.349 in 2024 (P = 0.001). The map was processed on QGIS and Geoda.

In 2013, the bivariate Moran’s Global Index indicated a positive spatial dependence between dengue incidence and average temperature (0.141; P = 0.001). This suggests that municipalities with higher average temperatures tended to cluster with higher incidence rates (Figure 1). In 2024, the index was negative (−0.089; P = 0.001). This result indicated an inverse spatial correlation, suggesting that higher incidence was observed in areas that have historically been classified as cooler by Köppen-based averages (Figure 1). It is imperative to acknowledge that these findings are exploratory and should be interpreted with caution because temperature alone cannot capture the complexity of dengue transmission. The observed patterns may have been influenced by the distribution and interaction of various determinants, including social vulnerability, mobility, urban infrastructure, vector control capacity, and population immunity.

The results are consistent with an epidemiological shift marked by the expansion of dengue into temperate areas of Brazil, Southeast Asia, South America, and Sub-Saharan Africa^2^. Municipalities with high incidence in 2024 demonstrated spatial dependence even in regions previously characterized by colder climates (Figure 1). This observation aligns with reports by Gurgel-Gonçalves, Oliveira, and Croda^3^ and supports the findings reported by Duarte et al.^4^.

These patterns may reflect increasingly favorable ecological conditions for *Aedes aegypti* ^9^ ^,^ ^10^, consistent with evidence showing that rising temperatures shorten developmental stages and increase vector density. Higher temperatures can accelerate all stages of the mosquito life cycle, contributing to greater population density and enhanced transmission potential^5^.

Duarte et al.^4^ found that average temperatures above 40°C or below 17°C were unfavorable because they impaired the dengue transmission dynamics. Furthermore, it was found that *Aedes aegypti* survival decreased at temperatures above 40°C or below 10°C^9^. An increase in the average monthly minimum temperature, generally around 21°C, and its association with increased dengue incidence^2^ was also identified in a study by Feng et al.^11^.

As climatic variables are increasingly incorporated into epidemiological analyses, studies should explicitly select and define how temperature-related variables will be used. This is of particular importance because alternative operationalizations, such as mean and/or extreme metrics, can differentially affect model behavior. These considerations highlight the need for integrated, interdisciplinary designs that collectively consider climatic exposure, epidemiologic dynamics, and contextual covariates to reduce confounding and enhance interpretability. Moreover, the limited availability of timely and interoperable meteorological data continues to constrain more robust, higher-resolution analyses. Regarding the 2024 dengue epidemic in Brazil, universal approaches for the surveillance and control of *Aedes aegypti* have gained prominence, including risk stratification and the use of ovitraps for entomological monitoring. Additionally, new approaches, such as the gradual release of *Wolbachia*-infected male and female mosquitoes, larvicide-disseminating stations, sterile insect techniques via irradiation, and dengue vaccination, have been noted^3^.

When defining strategies for each municipality, decision-makers should consider environmental, sociodemographic, and health system contexts^3^ ^,^ ^9^ while prioritizing sanitation and safe water storage. At the municipal level, integrating local climatological indicators with surveillance data can support territory-specific risk detection and strengthen early warning and climate adaptation plans.

The Köppen methodology^12^ and its proposed updates for Brazil provide standardized climatic profiles for epidemiological research. However, incomplete national meteorological records and limited access to updated operational temperature data pose methodological constraints for national and regional analyses.

We acknowledge that reliance on long-term average-based classifications, such as the Köppen system, may not capture recent thermal variability or extreme events, potentially leading to exposure misclassification. Despite this limitation, the Köppen classification remains one of the most standardized and internationally comparable climatic frameworks, justifying its application in large-scale ecological studies.

If nationwide health data can be systematically organized and made publicly available through the Department of Information and Health Informatics of Brazil’s Unified Health System, similar efforts should be directed toward climatic data produced by the National Institute of Meteorology (INMET), the agency responsible for producing meteorological information in Brazil, given its relevance to climate-sensitive health outcomes.

The systematic organization and integration of meteorological data into interoperable databases linked to health services would strengthen climate-informed surveillance and public health planning. Therefore, government investment in reliable, accurate, and timely climate data is essential to meet the needs of the Brazilian Unified Health System (SUS) and society.

InfoDengue (Fiocruz/FGV) is a relevant initiative that operates as a nationwide early warning (nowcasting) system for dengue, chikungunya, and Zika, combining routine surveillance data with Copernicus ERA5 meteorological indicators (especially temperature and humidity) to generate weekly municipal-level transmission risk alerts^8^.
